# High-Capacity Adenoviral Vectors: Expanding the Scope of Gene Therapy

**DOI:** 10.3390/ijms21103643

**Published:** 2020-05-21

**Authors:** Ana Ricobaraza, Manuela Gonzalez-Aparicio, Lucia Mora-Jimenez, Sara Lumbreras, Ruben Hernandez-Alcoceba

**Affiliations:** Gene Therapy Program. University of Navarra-CIMA. Navarra Institute of Health Research, 31008 Pamplona, Spain; aricobaraza@unav.es (A.R.); gamanuela@unav.es (M.G.-A.); lmora.1@alumni.unav.es (L.M.-J.); slumbreras@alumni.unav.es (S.L.)

**Keywords:** adenovirus, high-capacity adenovirus, helper-dependent, gutless, gene therapy, vector, gene correction

## Abstract

The adaptation of adenoviruses as gene delivery tools has resulted in the development of high-capacity adenoviral vectors (HC-AdVs), also known, helper-dependent or “gutless”. Compared with earlier generations (E1/E3-deleted vectors), HC-AdVs retain relevant features such as genetic stability, remarkable efficacy of in vivo transduction, and production at high titers. More importantly, the lack of viral coding sequences in the genomes of HC-AdVs extends the cloning capacity up to 37 Kb, and allows long-term episomal persistence of transgenes in non-dividing cells. These properties open a wide repertoire of therapeutic opportunities in the fields of gene supplementation and gene correction, which have been explored at the preclinical level over the past two decades. During this time, production methods have been optimized to obtain the yield, purity, and reliability required for clinical implementation. Better understanding of inflammatory responses and the implementation of methods to control them have increased the safety of these vectors. We will review the most significant achievements that are turning an interesting research tool into a sound vector platform, which could contribute to overcome current limitations in the gene therapy field.

## 1. Introduction

### 1.1. General Characteristics of Adenoviruses (AdVs)

The *Adenoviridae* family comprises a wide group of human and animal viruses sharing functional, genetic, and structural characteristics [1]. Traditional classification in serotypes, based on immune cross-reactivity, is being substituted by sequence homology for the identification of new AdV types [2]. Human adenovirus type 5 (HAdV5), belonging to the *Mastadenovirus* genus, is the adenovirus most frequently adapted as gene therapy vector, and we will use it as a paradigm for the description of general characteristics. However, the entire *Adenoviridae* family offers a rich source of members [3] whose peculiarities are being recently exploited for therapeutic purposes (Table 1). In general, the viral particles consist of a linear double-stranded DNA genome ranging from 26 to 46 Kb in length (36 Kb in the case of HAdV5), embedded in core proteins and surrounded by an icosahedral capsid [4]. Each vertex of this outer structure contains five units of a protein called penton, which act as an anchor for the trimeric protein fiber. The most abundant capsid protein is the hexon, which together with other structural proteins forms the facets of the icosahedron. The average size of viral particles is 100 nm in diameter. The C-terminal portion of the fiber (knob) interacts with the primary receptor in cells [5], allowing an initial immobilization of the particle in the cell surface, which facilitates further interaction between the penton base and integrins [6], as well as binding of fiber shaft to heparan sulphate proteoglycans. These can be considered secondary receptors, which allow infection of cells lacking the primary receptor, albeit at a lower rate. This is the reason why most AdV types present a wide cellular tropism. This fact, together with the possibility of obtaining the virus at high titers, determines that few cells are completely refractory to AdV infection, at least in vitro. However, AdV types using Coxsackie and Adenovirus Receptor (CAR) as primary receptor show preference for epithelial cells, whereas other types such as HAdV35 use CD46 or desmoglein-2, and are able to infect hematopoietic cells (Table 1) [7]. Upon cell attachment, internalization of the virion is activated through chlatrin-mediated endocytosis [8]. A programmed disassembly of the capsid is required for further progression of particles in their journey to the cell nucleus [9]. Of note, this is an active and very efficient process which largely contributes to the high efficacy of transduction [10]. The endocytic vesicles are disrupted owing to the release of protein VI from the capsid, avoiding lysosomal destruction of the virus [11]. The particles are then transported to the nuclear pore using the microtubular complexes, and the DNA together with some core proteins are finally introduced into the nucleus [12]. It is estimated that 40% of internalized particles complete this process in less than 2 h. Importantly, the double-stranded genome is ready to be transcribed once inside the nucleus, which ensures a rapid and efficient expression of vector-encoded proteins. Wild type AdVs follow a lytic cycle in permissive hosts, completing the steps of early viral genes expression, genome replication, late viral genes expression, genome encapsidation, and particle maturation inside the nuclei. At the end, nuclear and cytoplasmic membranes are disrupted and up to 10,000 new virions are released 48–72 h after infection in highly permissive cells. Most wild type AdVs cause self-limited infections in their respective host species, affecting predominantly the respiratory tract, eyes, and digestive tract, depending on the serotypes (Table 1) [13]. However, human AdVs are severe pathogens in immunocompromised individuals such as patients undergoing bone marrow or solid organ transplantation [14]. In these cases, AdV is found in circulation and shows its capacity to infect internal organs such as liver. Interestingly, the origin of these infections is usually the reactivation of viral reservoirs present in the gut [15], which demonstrates the possibility of latency or chronic, sub-clinical persistence of the virus.

### 1.2. AdVs as Therapeutic Agents: Versions and Evolution

**Figure 1:**
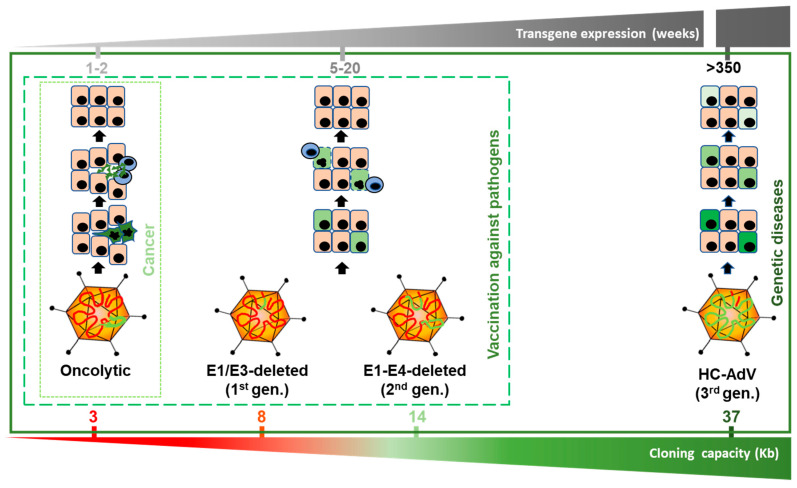
Versions of adenoviral vectors and potential therapeutic applications. The size range allowed for genome packaging is between 28 and 38 Kb. Oncolytic adenoviruses retain most of the viral genome, including the E1 region, which is required for replication. They can accommodate up to 3 Kb of exogenous DNA if the E3 region is partially deleted. Since they replicate their genomes and cause the lysis of infected cells, transgene expression is very intense but transient. Among replication-deficient vectors, E1/E3-deleted (1st generation) versions can harbor up to 8 Kb. This capacity can be extended up to 14 Kb if the E2 and E4 regions are also deleted (second generation). Although these vectors do not cause direct destruction of infected cells, cytotoxic immune responses against them limit the stability of transgene expression. Apart from vaccination strategies, E1/E3-deleted vectors are still a widely used research tool for in vitro and in vivo gene transfer. High-capacity adenoviral vectors (HC-AdVs) (third generation) only retain short non-coding regions from the AdV genome (ITRs and ψ signal), which leaves a cloning capacity of 37 Kb. The lack of viral gene expression in transduced cells reduces cellular immune responses and allows long-term transgene expression, which decreases slowly as the cells are renewed. HC-AdVs are suitable for all in vivo applications, including gene supplementation and gene correction for monogenic diseases. The indicated duration of transgene expression is based on liver-directed transduction in NHP, but it can be different in other hosts and tissues. In vector genomes, viral DNA is represented in red and exogenous DNA in green (including expression cassettes and/or DNA templates, and stuffer DNA).

AdVs can be modified to exploit different properties, giving rise to specialized agents (Figure 1). The lytic cycle can be used to destroy cancer cells (oncolytic adenoviruses, OAV). To this end, replication of the virus should be properly modulated by transcriptional control of the early viral genes (particularly E1A) or ablation of viral functions that are dispensable only in cancer cells [17]. OAVs can incorporate transgenes in order to enhance their therapeutic effect, giving rise to the “armed” OAVs, also called replication-competent vectors. In contrast, when the primary objective is to express transgenes in the target cells, the replicative potential of AdV should be abolished in order to prevent the destruction of cells. This is accomplished by removal of the E1 region, which is complemented *in trans* in the packaging cells during vector amplification. The E3 region, which is dispensable for the amplification of AdV in cell culture, is also removed in most cases to increase the cloning capacity up to 8 Kb. These first-generation, E1/E3-deleted AdV vectors (FGAdV) demonstrated high transduction efficacy in cell cultures and animal models, and its clinical translation raised high expectations [18]. Further deletion of viral genes such as E2 and E4, owing to the development of the corresponding trans-complementing cells, increased the cloning capacity of these second generation AdV vectors (Figure 1). However, the presence of a substantial proportion of the wild type genome leads to a residual expression of viral proteins in the transduced cells, and elicits cytotoxic immune responses against them, limiting the duration of transgene expression in immune-competent hosts [19]. Therefore, the current application of these early generation vectors is limited to vaccination strategies [20], apart from their use as research tools for cell culture transduction. It was only when all viral coding sequences were completely removed (3rd generation, “*gutless*” vectors) that cellular immune responses against transduced cells were dampened and long-term transgene expression was achieved in vivo. Seminal studies in non-human primates (NHP) demonstrated that a single intravenous administration can sustain secretion of therapeutic proteins from the liver for several years [21,22]. In these vectors, only the inverted terminal repeats (ITR), the packaging signal (Ѱ), and in some cases a short non-coding region from the right end of the viral genome, are maintained [23]. These small sequences are required for genome replication during vector production, encapsidation of genomes, and maintenance of genome stability, respectively. The cloning capacity of these vectors is extended to 37 Kb, which justifies their denomination as high-capacity adenoviral vectors (HC-AdVs). Since the size of expression cassettes is usually smaller, HC-AdVs need to incorporate stuffer DNA in their genomes in order to reach the minimal size for stable packaging, which is close to 28 Kb in the case of HAdV5 [24]. Of note, packaging cells expressing all viral genes are not viable, as discussed below. Therefore, trans-complementation is usually achieved using a helper virus (HV), which is a specialized E1/E3-deleted AdV vector. This explains the alternative denomination as helper-dependent AdV vectors (HD-AdV). In the next sections, we will summarize and discuss different aspects of HC-AdVs, with special attention to the therapeutic possibilities they offer and the current challenges in the field.

## 2. Production of HC-AdVs

The removal of all viral coding genes dictates the unique properties of HC-AdVs in terms of stability of expression and cloning capacity, which differentiate them from early AdV vector versions. The downside is a greater complexity of the production procedures, because stable expression of all adenoviral genes in packaging cells, in the pattern and amount required for trans-complementation, is not feasible. Therefore, most methods rely on the use of HVs. The main differences among them are the strategies used to avoid HV contamination in the final HC-AdV preparation, as described below.

### 2.1. Viral Rescue and Amplification

The first method for HC-AdV production dates back to 1996, when Kochanek et al. prepared a plasmid containing 28 Kb of non-viral DNA flanked by AdV ITRs, with the packaging signal in one end [25]. The HV was a FGAdV with a 91 bp deletion in the packaging signal, so it would have a competitive disadvantage for encapsidation. When the plasmid was transfected in HEK293 cells and then cells were infected with the HV, they could rescue a small amount of HC-AdV. After several rounds of co-infection in a growing number of cells, the vector was purified by ultracentrifugation in a density gradient. The final contamination with HV was in the range of 1%, but production of HV was problematic because of the inefficiency of packaging. Later, Parks et al. introduced the concept of Cre-mediated excision of the packaging signal [26]. When this sequence is flanked by loxP sites in the HV and the cells express the recombinase, high yields of HC-AdV with less than 0.1% HV contamination can be routinely obtained. Apart from the efficient removal of the packaging signal, an important advantage of this methods is the easy production of the HV in standard HEK293 cells. Although similar results were obtained when the FLP/frt system was used [27], the Cre/loxP system is currently the gold standard, and it is the basis for most improvements developed since then, as described below. The HV is usually an E1/E3-deleted vector, but restoration of the E3 region has been described to increase its helper efficacy. On the other hand, deletion of the E2 region may increase the safety of HVs, although special packaging cells complementing the E1 and E2 genes are required [28]. During HC-AdV amplification, the shut-off of protein synthesis in the cell, imposed by the virus, limits the availability of the Cre recombinase. This is the moment of highest demand for the removal of packaging signal in a growing number of HV genomes [29]. To avoid this limitation, a self-inactivating HV was developed in which the recombinase is inserted in its own genome [30]. Owing to a drug-inducible system and the use of the MerCreMer fusion protein [31], cleavage of ψ can be modulated and this HV can be produced in HEK293 cells. In contrast, this sequence is efficiently cleaved when the expression of MerCreMer is stimulated by doxycycline, and the addition of 4-hydroxy-tamoxifen allows the access of the protein to the nucleus. Increasing the difference in genome size between HV and HC-AdV facilitates the separation of the particles by density, and inverting the orientation of the packaging signal in the HV reduces the risk of productive recombination with the HC-AdV genomes [32]. Deletion of the pIX gene decreases the packaging capacity of HAdV to 35 Kb. If the size of the HV exceeds this limit and harbors this deletion, it can only be produced in specialized HEK293 cells expressing pIX. This phenomenon can be exploited to reduce HV contamination [33]. Flanking the HV packaging signal by attB/attP sequences produces a delay in encapsidation, which can be used to reduce contamination [34]. Other methods rely on the incorporation of the HV genome in other vectors such as Baculovirus [35] or herpes simplex virus (HSV) [36]. More recently, a HV-free method has been described, in which all trans-complementing genes are provided by transfection of a plasmid devoid of packaging signal, in several steps of amplification [37]. This procedure is reminiscent of the initial stages of amplification in pioneering protocols [38], but the new method relies on co-transfection of a plasmid encoding the AdV pre-terminal protein (pTP) to enhance vector yield. This result challenges the notion that TP should be covalently fused to both genome ends in order to promote genome replication [39]. The suitability of this procedure for large-scale production awaits confirmation.

### 2.2. Purification

In principle, HC-AdVs can be purified the same as any other AdV. Ultracentrifugation in CsCl density gradients is the traditional method, followed by desalting by size exclusion chromatography (sepharose columns) or dialysis [40,41]. However, iodixanol may have advantages compared with CsCl. On the one hand, it is more biologically compatible and requires shorter centrifugation times. On the other hand, it can preserve particle infectivity during the purification process, and provides better separation of particles with small differences in genome size. In fact, reduction of HV contamination from 2.5 to 0.03% has been reported after two iodixanol purification steps [42]. In principle, these methods allow to discriminate empty and incomplete viral particles from particles containing full vector genomes, improving (reducing) the total to infectious particle ratio. This is especially relevant for HC-AdVs, since crude lysates often present very high ratios. When the difference in genome sizes between HC-AdV and HV is sufficient, selecting the correct fraction can also reduce HV contamination [27]. Anion exchange columns and density gradients can be used sequentially to improve separation [42]. However, ultracentrifugation is not convenient for high-scale production and good manufacturing practices (GMP) adaptation. A combination of chromatographic methods based on capture antibodies, ionic exchange, size exclusion, hydrophobic interaction, and immobilized metal affinity columns has been described [43,44]. Methods that contribute to the enrichment in full vector particles are especially indicated for HC-AdV [45,46].

### 2.3. Quantification

Common to all viral vectors, the availability of standardized methods for precise quantification of HC-AdVs is an unmet need. The simplest way to determine the amount of particles (vp) in a purified HC-AdV preparation is based on the absorbance at 260 nm, usually performed after disruption of capsids by detergent (SDS) or enzymatic treatment [47,48]. Similar to other vectors such as those derived from adeno-associated virus (AAV), quantitative PCR can be used to determine the amount of viral genomes (vg) [49], which should provide information equivalent to the spectrophotometer. In both cases, the availability of certified standards could contribute to the reproducibility of results, the comparison of different vector batches, and the uniformity of data across laboratories [50]. In contrast with early generation AdV vectors, HC-AdVs are not replicative even in the packaging cells. Therefore, quantification of plaque forming units (pfu) or infectious units (iu) using end-point dilution methods or commercially available kits is not possible. For determination of iu, permissive cells exposed to the vectors can be lysed a few hours later and viral genomes are quantified by PCR [49,50]. Although this method is more restrictive than direct PCR of particles and provides a closer estimation of transduction potency, it is difficult to standardize, and the values are always relative to the cell line and the culture conditions employed. However, iu quantification can explain apparent inconsistencies in the performance of different vector batches. Only when the production process is perfectly standardized, an equivalent ratio between total and infectious particles can be assumed. This ratio is usually higher in HC-AdVs compared with OAVs or FGAdV vectors, and differences have also been reported among AdV vector platforms. For instance, vectors derived from canine adenovirus type 2 (CAV-2) present ratios of less than 3:1 [51], whereas ratios of 10:1 or higher are common for human vectors, although it can be due to the specific cell lines employed for quantification. Comparing the performance of different vectors such as those derived from AAV and AdV is even more complicated. The few articles taking this challenge usually report the dose of AdV using iu and vp, whereas AAV are quantified in vg [52]. However, a relevant comparison should take into account different parameters such as the balance between safety and efficacy, feasibility of production, and the amount of vector genomes in the target organ needed for the therapeutic effect.

## 3. Immune Responses and Other Host–Vector Interactions

All HC-AdV interactions with the extracellular milieu are common to the parental AdV type used to develop the HV, unless specific capsid modifications have been implemented. This means, for instance, that HC-AdVs derived from the prototypic HAdV5 will bind with high affinity to erythrocytes in circulation, through direct binding of fiber to the primary CAR receptor, or via natural antibodies to complement receptor CR1 [53]. It is not entirely clear whether this is a barrier for AdV dissemination or if the virus can use these cells as carriers. Vitamin K-dependent coagulation factors such as FVII, FIX, and FX bind to the hexon of many AdV types (including HAdV5). This is a critical factor for transduction of hepatocytes [54], and it can shield the particles from natural IgM antibodies and from the complement system, favoring the function of intravenously administered vectors [55].

In the same vein, HC-AdVs elicit innate and humoral adaptive immune responses similar to other AdV vectors [56]. Of note, inflammatory responses against AdV are largely responsible for the toxicity observed when these vectors are administered systemically at high doses [57]. A dose-dependent elevation of cytokines such as interleukin-6 (IL-6) has been documented in animal models and patients [58,59], and a similar scenario is expected in the case of HC-AdVs [60]. In the vast majority of cases, this is a self-limited event. However, the death of a patient in a pioneering clinical trial 4 days after receiving 3.8 × 10^13^ vp of an early generation AdV vector [59] is a constant reminder that cytokines should be monitored. New methods to counteract inflammatory mediators such as IL-6 and tumor necrosis factor alpha (TNFα), including monoclonal antibodies, should be available to guarantee the safety of clinical trials based on systemic administration of these agents [61]. It is reassuring to see that after more than 500 clinical trials involving AdV vectors and OAVs, no more vector-related life-threatening adverse effects have been reported. Although clinical experience in specific populations such as young children and severely immunocompromised patients is limited, an increase in immune-related adverse effects is not expected. In the latter group, the risk of OAV replication in normal tissues should be carefully evaluated.

In contrast with the aforementioned considerations, vector-related cellular immune responses against transduced cells are attenuated in the case of HC-AdVs compared with other AdV vectors [62,63,64]. The rapid degradation of incoming capsids and the lack of viral genes encoded in the vector genome reduce the possibility of presenting highly immunogenic viral epitopes in the surface of the cells. AdV-specific T-cells responses can be stimulated after intravenous administration of HC-AdVs in different animal models [56,65], but in contrast with early generation AdV vectors [66], a biphasic elevation of liver transaminases is not observed [56,67]. This is consistent with a dose-dependent inflammatory response leading to early and transient liver damage, which is not followed by cytotoxic immune responses against transduced hepatocytes (Figure 1). Importantly, the pre-existing anti-AdV humoral and cellular immune responses do not compromise the stability of transgene expression in mice treated with HC-AdVs [62,64], increasing the prospects for successful application of these vectors in humans. However, AdV-specific CD4 T cells are detected in a high proportion of individuals [68], and the possibility of cytotoxicity against transduced cells harboring incoming viral particles cannot be ruled out. Since the predictive value of animal models toward vector-related immune responses is limited, carefully designed clinical trials should investigate this phenomenon. Experience with AAV vectors suggests that rapid or prophylactic corticoid treatment could overcome immune-related loss of transgene expression [69].

## 4. Genome Stabilization in Transduced Cells

HC-AdV particles penetrating into mammalian cells are transported through microtubules and introduce their genome in the nucleus, following the highly efficient pathway common to all AdV vectors [8]. During this process, the incoming particle components and the vector DNA can be detected by pattern recognition receptors (PRRs) such as Toll-like receptors (TLRs) and nucleic acid sensors, triggering cellular antiviral defense mechanisms (type I interferons (IFNs), TNFα and other cytokines, depending on the cell type) [70,71,72]. Compared with the wild type infectious cycle, the absence of viral replication in HC-AdVs reduces the activation of antiviral defenses, but on the other hand the lack of E3 and E4 genes eliminates the natural mechanisms that AdV has developed to counteract these responses [73,74,75]. It is difficult to determine to what extent the antiviral pathways reduce the efficacy of transgene expression by DNA degradation, transcriptional repression, or other mechanisms. However, the net balance of these early vector-cell interactions is, in a relevant proportion of cells, the persistence of HC-AdV genomes in an episomal state [76]. The kinetics of transgene expression in vivo, in organs such as the liver, is compatible with permanent presence of transcriptionally active genomes during the entire lifespan of transduced cells, when the appropriate promoters are used [22]. During cell division, HC-AdV genomes are more stable than dsDNA fragments transfected by non-viral methods [77], suggesting specific mechanisms of retention. Although a low proportion of chromosomal integrations have been detected, this is a random process inherent to the presence of linear DNA in the nucleus, and it cannot account for the stability of transgene expression in vivo [78]. AdV genomes enter the nucleus still bound to core proteins, and as soon as 1 h after infection, both protein VII and cellular histones are associated with the viral DNA. In the absence of replication, the predominant histone found in vector genomes is H3.3, which progressively replaces protein VII [79]. The process is dependent on the chaperone HIRA [80], and this chromatinization seems to be important for the stabilization of transgene expression. It is not surprising that sequences contained in the vector genome determine its epigenetic status and exert a strong influence on the expression cassette. The cloning capacity of HC-AdVs allows the incorporation of human genomic fragments partially mimicking the chromosomal regions of interest. Therefore, the so called “stuffer” DNA may play a relevant role as part of the expression cassette [81]. Genome persistence at the cellular level is a critical event, but the interplay between cellular, tissue, and systemic factors has a major impact on the stability of transgene expression in vivo. For instance, an initial period of high expression followed by a decline and then long-term stabilization has been reported in some studies using intravenous administration of HC-AdVs [21,77,82]. This may be due to the initial transduction of several populations of cells in the liver, each one with a different turnover (long-lasting hepatocytes versus Kupffer cells, etc.). The same could apply to glial versus neuronal populations in the brain [62]. Of note, this phenomenon can lead to apparent disappearance of transgene expression if the initial intensity is low, depending on the sensitivity of detection methods [83]. Finally, the influence of the transgene product should be taken into account. These factors may explain some differences observed when the vectors are administered to rodents, dogs, macaques, and baboons at different ages. Importantly, remarkable stability has been observed in adult NHPs when the transgene encodes an endogenous protein, which could be detected in serum more than 7 years after initial vector administration [22].

## 5. Beyond HAdV5: Expanding the Repertoire of HC-AdVs

HAdV5 is a robust platform for the construction of HC-AdVs. Binding of capsids to the primary receptor CAR, and the interaction with secondary receptors such as integrins and HSPG, allow efficient infection of a variety of cell types, especially those of epithelial origin [8]. In addition, the length of its fiber and the interaction with blood proteins determine a marked liver tropism upon intravenous administration [54,84]. However, other HAdV5 features are not so favorable, such as the high frequency of neutralizing antibodies (NAbs) in adults [85,86] and the massive uptake by resident macrophages in the liver (Kupffer cells), which contributes to inflammation and reduces hepatocyte transduction [87,88,89]. In addition, HAdV5 is relatively inefficient infecting hematopoietic cells, and some tumors may reduce the expression of CAR [90,91]. Luckily, the *Adenoviridae* family provides a wide repertoire of members with specific properties matching many therapeutic needs (Table 1) [92,93]. Moreover, new capsid variants can be obtained by pseudotyping [94,95,96,97,98], rational design [99,100,101], or forced genome recombination followed by in vitro or in vivo screening [102]. Of note, in most cases the same HC-AdV genome can be incorporated in different capsids depending on the HV employed, which means that all the technology developed to target FGAdV and oncolytic vectors can be readily applied to HC-AdVs [103,104]. Producing a collection of antigenically distinct vectors would enable efficient re-administration, if needed, or heterologous prime-boost vaccination regimes. Finally, vectors can be coated with a variety of polymers in order to change their properties (reviewed in [105,106]). This can reduce their immunogenicity and protect particles form NAbs. In many cases, masking AdV epitopes results in loss of infectivity, which can be restored and re-directed to specific cell populations by ligand attachment into the shield [107]. Chemical coating can be facilitated by discrete genetic modification in the capsid. For instance, alanine to cysteine mutation into the hypervariable region 5 (HVR5) of HAdV5 allows efficient attachment of polyethylene glycol (PEI), which modulates hepatocyte transduction and reduces uptake of capsids by Kupffer cells [108].

Members of species B HAdV such as HAdV35 bind to CD46 as a primary receptor instead of CAR, which allows them to infect cells of hematopoietic origin, including hematopoietic stem cells (HSC) [7]. The construction of chimeric HVs displaying the HAdV35 fiber in the context of the standard HAdV5 backbone made it possible the advent of HC-AdVs with this expanded tropism [109]. HAdV35 fiber knob mutants with enhanced affinity for CD46 (35++) have been selected from an *E.Coli* expression library, and vectors displaying the selected variants showed improved transduction of CD46^+^ cells in vivo, with lower liver sequestration [94]. Coupled with other strategies for genome integration, these vectors are opening unprecedented opportunities in genetic and acquired diseases, as will be discussed in the next sections. Other HAdV types such as HAdV6 show reduced uptake by Kupffer cells. This property can be conferred to HAdV5-based vectors by swapping the hexon hypervariable regions between both types of vectors [95]. The chimeric 5/6 HC-AdV vector showed enhanced liver transduction and lower inflammatory reactions compared with equivalent vectors derived from HAdV5, as expected [96]. Interestingly, it was also superior to those derived from HAdV6, probably because the longer shaft of HAdV5 fiber is favorable for hepatocyte infection. The modularity of AdV components and the compatibility of them among different members of the family allow a vast number of potential combinations.

AdV derived from other host species are being exploited for therapeutic purposes, and some of them have been modified as HC-AdVs (Table 1). Apart from other specific properties, these agents have the advantage of low seroprevalence in humans, which facilitates their clinical application. However, cross-reactivity of NAbs, and especially T cells, have been reported between HAdV5 and certain chimpanzee AdVs, such as type 6 (ChAdV6 or AdC6) and ChAdV7 [110,111]. This unfavorable event seems more unlikely in AdVs isolated from more distant species such as bovine and porcine [112]. The downside is that these viruses usually require the development of dedicated production systems, in particular packaging cells. Regarding safety, it should be taken into account that HC-AdVs present the highest degree of attenuation, thus avoiding the risk of unexpected virulence in humans. The CAV-2 presents a strong dependence for CAR in order to infect cells, which implies that in the brain it shows preferential tropism for neurons versus glial cells [113]. This may reduce inflammatory responses and contribute to the accessibility of the target cells for many genetic diseases affecting the brain. In addition, CAV-2 vectors show an efficient axonal transport from neurites to the soma, because their intracellular trafficking mechanism relies on a vesicular pathway instead of the association of naked particles to the cellular microtubular network [114]. It is believed that this pathway protects the capsid from degradation in their journal through long axons. Therefore, HC-AdVs based on CAV-2 have been developed for the treatment of neurological diseases [115], as recently reviewed [116].

## 6. Therapeutic Applications of HC-AdVs

### 6.1. Gene Supplementation in Monogenic Diseases

Since AdV vectors are non-integrative, transgene expression will only be stable if the target cells have a low turnover rate, unless artificial mechanisms are designed to sustain genome replication or chromosomal integration [117]. In concordance with the natural tropism of the standard HAdV5-based vectors, HC-AdVs have been applied mainly to transduce the liver.

#### 6.1.1. Liver-Directed Gene Supplementation

Gene expression from hepatocytes has therapeutic interest not only for hepatic and metabolic diseases, but also for secretion of proteins into circulation. Hemophilia has been considered a suitable target for liver-directed gene therapy from the beginning of this field, because coagulation factors are naturally produced and secreted from this organ. Transduction of all hepatocytes is not required to obtain a therapeutic effect, as far as the circulating levels of the proteins reach a certain threshold. In general, 5% normal values are needed to convert a severe hemophilia into a mild disease, and 30–50% should be obtained for complete clinical normalization [118]. Hemophilia A was an obvious indication for HC-AdVs because the 7 kb-long coagulation factor VIII cDNA (*F8*) exceeds the cloning capacity of AAVs. Pre-clinical studies in mice showed restoration of circulating FVIII at therapeutic levels using a vector encoding human *F8* under the control of the albumin promoter [119]. Following safety assessment in mice and dogs [120], this strategy moved quickly into the clinic. In 2001, the first patient treated intravenously with 4.3 × 10^11^ vp/Kg of the vector showed initial signs of efficacy (increase from less than 1% to 3% FVIII levels in serum), according to sponsor´s press releases. However, the appearance of acute thrombocytopenia, increased liver transaminases and elevation of IL-6 prevented the enrollment of more patients in this cohort. Since safer doses of this vector had low therapeutic possibilities, the trial was not continued, and full description of this case is not available in the scientific literature. Although only speculative, one possible explanation for this outcome is a relatively high HV contamination combined with high total to infective particle ratio during mass production of the vector, since the method available at that time was not optimal [25]. In addition, expression of the prokaryotic *LacZ* gene from HV-transduced hepatocytes could contribute to liver damage and immunogenicity. Subsequent pre-clinical developments, including improvements in the production methods, showed therapeutic benefit and moderate, dose-dependent toxicity in stringent models such as hemophilic dogs [67,82,121]. The failure to maintain high levels of FVIII in circulation was mainly attributed to the intrinsic immunogenicity of this protein. In fact, neonatal administration of the vectors achieved tolerance and improved the performance of re-administration in adults, at least in mice [122]. The use of liver-specific promoters also reduces the possibility of transgene expression in antigen-presenting cells [123]. However, no further clinical trials were performed, in part because of the development of efficient AAV vectors carrying shorter versions of the *F8* cDNA [124,125].

Gene therapy approaches for hemophilia B followed a similar pattern. In this case, the relatively small size of the mutated gene (*F9*, cDNA 1.5 Kb) allows the use of AAV vectors without transgene engineering, which has expedited its clinical translation [118]. Other circumstances favoring the success of gene therapy for hemophilia B, irrespective of the choice of vector, include the efficient secretion of the therapeutic protein from the liver and the lower frequency of alloantibody development in patients, compared with hemophilia A [126]. HC-AdVs expressing *F9* under the control of liver-specific promoters have shown excellent preclinical results in murine hemophilia B models [127]. Canine models show higher variability, and some cases of premature transgene expression shutoff have been reported for unknown reasons [128]. However, further work achieved sustained therapeutic levels of clotting FIX in this stringent model [129]. Importantly, studies in NHP confirmed the stability of transgene expression previously observed using other transgenes such as α-1 anti-trypsin (A1AT) and α-fetoprotein [21,58]. In order to enhance liver transduction while reducing systemic exposure to the vector, a HC-AdV encoding human FIX was injected in rhesus macaques through the hepatic artery with transient balloon occlusion of the inferior vena cava. The maximal dose reported (1 × 10^12^ vp/Kg) was needed to guarantee sustained therapeutic levels of the clotting factor for more than 2 years [130]. No serious adverse effects were observed, but further studies are needed to determine the maximal tolerated dose and therapeutic range of this approach. Of note, clinical experience indicates that 6 × 10^11^ vg/Kg of an E1-E4-deleted AdV administered through the portal vein caused a severe inflammatory syndrome in one human subject [59].

Another example in which transgenes expressed from the liver and secreted into circulation can be therapeutic is A1AT deficiency (A1ATD). HC-AdVs encoding A1AT were among the first vectors of this class to be tested in animal models, including NHP [21,131]. In fact, A1AT has been used as a reporter gene in the early evaluation of HC-AdVs [132]. In all cases, efficient and long-lasting expression has been demonstrated. However, the therapeutic potential has not been evaluated in A1AT-deficient models. Since the liver is also affected in this disease because of the aggregation of the mutated protein, full recovery requires simultaneous transgene expression and inhibition/disruption of the endogenous gene. This challenge is now feasible owing to new gene editing tools, and HC-AdVs are a suitable vector platform, as will be discussed in Section 6.2.

Systemic administration HC-AdVs have shown therapeutic effect in different models of dyslipidemia, in which elevated and sustained levels of therapeutic proteins are needed in circulation. A pioneer study demonstrated the lifelong correction of cholesterol levels in Apo E-deficient mice upon a single intravenous administration of HC-AdVs, especially when they incorporate Apo E gene and regulatory sequences in the genomic context [133]. Vectors encoding Apo AI achieved elevation of high-density lipoproteins (HDL) and reduction of low density lipoproteins (LDL), as well as prevention of atherosclerotic lesions in Apo AI-deficient mice [63,134]. Reduction of cardiovascular risk was also demonstrated in Apo E and LDL receptor-deficient mice [135,136]. Apart from liver-directed gene therapy, direct vascular wall transduction is being investigated for reversion of atheromatous plaques. Sustained expression of endothelial cells has been demonstrated upon intra-arterial delivery of HC-AdVs. Using this approach, significant improvement has been observed in high fat diet-fed rabbits [137]. Co-expression of Apo AI and the ATP-binding cassette subfamily A, member 1 (ABCA1) is feasible using HC-AdVs, and this combination can improve the cholesterol efflux from endothelial cells [138]. Reduction of cholesterol and cardiovascular protection was also observed in LDL receptor (LDLR)-deficient mice treated with a vector encoding LDLR. Interestingly, a vector encoding the very low density lipoprotein (VLDL) receptor gene obtained a partial correction of cholesterol levels, probably because it can only restore uptake of Apo E, but not Apo B100-containing lipoproteins [139]. Later on, experiments carried out in rhesus macaques heterozygous for a LDLR mutation highlighted the potential limitations of this approach in the clinical setting, and suggested potential solutions [140]. First, dose-dependent toxicity associated with inflammatory responses was confirmed following intravenous injection of the vector. Taking into account that diseases such as LDLR deficiency require high levels of transgene expression to obtain a therapeutic effect (at least 50% normal values), the therapeutic index of intravenous administration is too narrow. This problem was circumvented by an optimized balloon occlusion method, which decreased the systemic exposure to the vector and reduced 5-fold the therapeutic threshold (1 × 10^12^ vp/Kg). The second serious concern was an elevation of transaminases (ALT) observed 2 months after treatment. Although the increase was mild and transient, it was followed by a reduction and virtually disappearance of the therapeutic effect (rebound of cholesterol levels to pre-treatment values). In principle, immune responses against the transgene were discarded because the vector expressed the rhesus monkey LDLR cDNA in haploinsufficient animals, and it was under the control of the liver-specific phosphoenol pyruvate carboxykinase (*PEPCK*) promoter. Although no T-cell responses could be detected against adenoviral proteins, this phenomenon is reminiscent of the situation observed in humans treated with AAV vectors [141]. It remains to be tested if the same management (short course of corticoid treatment) will be effective in the case of HC-AdVs, if needed.

Primary hyperoxaluria type 1 (PH1) is caused by defects in alanine:glyoxylate aminotransferase (AGT), resulting in systemic elevation of oxalate and accumulation of calcium oxalate precipitates in the kidney and other organs. This disease is considered a suitable target for liver-directed gene therapy, since there is clinical evidence that liver transplantation (combined with kidney transplantation in most cases) is therapeutic [142]. However, initial preclinical studies indicated that a high percentage of hepatocytes should be transduced in order to obtain a therapeutic effect. In a mouse model of PH1, a HC-AdV expressing AGT under the control of the liver-specific PEPCK promoter showed a dose-dependent reduction of oxalate in serum [143]. Normal values were only obtained when at least 80% of hepatocytes expressed the transgene (at a dose of 5 × 10^12^ vp/Kg of vector), suggesting that PH1 is still a challenge for current gene therapy technologies.

An equivalent vector was employed for the treatment of carbamoyl phosphate synthetase 1 deficiency (CPS1D). HC-AdVs are especially indicated for the treatment of this urea cycle disorder, since the size of the *CPS1* cDNA (4.5 Kb) makes it difficult to design efficient AAV vectors. In this case a high dose (5 × 10^12^ vg/Kg) was also needed for full protection from hyperammonemia in a mouse model [144]. Apart from this, clinical implementation of this approach is further complicated by the need to transduce the immature and rapidly growing liver of newborns. Indirect preclinical evidences generated in a hemophilia A mouse model suggest that HC-AdVs could be efficiently re-administered after a first neonatal dose [122], but this possibility needs confirmation in other animals and in humans.

For diseases requiring high levels of transgene expression, optimization of delivery routes and expression cassettes is crucial, as demonstrated for Crigler-Najjar syndrome type I in the Gunn rat model. The therapeutic dose of a HC-AdV encoding Uridine diphospho-glucuronyl transferase 1A1 (*UGT1A1*) was reduced from 3 × 10^12^ vp/Kg to 5 × 10^10^ vp/Kg when an enhancer from the *ApoE* gene was located in the 3’UTR of the transgene, and the vector was delivered by hydrodynamic injection [52]. Clinically compatible methods for enhanced liver transduction are being developed [145].

In acute intermittent porphyria (AIP), mutations in one copy of the porphobilinogen deaminase gene (*PBGD*) cause a reduction in the expression of the enzyme, which is involved in the heme synthesis pathway. Patients suffer attacks of severe abdominal pain and neurovisceral disturbances, which can be life-threatening and provoke progressive irreversible neuropathy [146]. Intravenous administration of a HC-AdV encoding human *PBGD* under the control of a potent liver-specific promoter (albumin enhancer linked to α1 anti-trypsin promoter) achieved correction of neurotoxic intermediate metabolites in a mouse AIP model [147]. Direct intra-hepatic injection obtained the same effect with a 7.5-fold reduction in the vector dose (2 × 10^11^ vp/Kg). However, experiments performed in macaques (*Macaca fascicularis*) showed that sustained expression of *PBGD* and persistence of vector genomes in the liver required intense immunosuppression [83]. It is not clear if this discrepancy versus previous studies using other HC-AdVs in baboons [22,58] is due to the transgene or the different NHP used.

For lysosomal storage disorders (LSD), transduced hepatocytes can become a stable source of therapeutic enzymes secreted into circulation and internalized by target cells through the mannose-6-phosphate receptor [148]. This is relevant in cases such as Pompe disease (defect of acid α-glucosidase, *GAA*), in which accumulation of glycogen in skeletal muscles, hearth and diaphragm plays a major role in the clinical manifestations. In fact, a HC-AdV expressing GAA under the control of liver-specific sequences (*PEPCK* promoter plus *ApoE* enhancer) achieved long-term correction of glycogen content in skeletal muscles [149]. Importantly, GAA levels in circulation and enzyme uptake in muscles were recapitulated in baboons using the balloon catheter occlusion technique (1 × 10^12^ vg/Kg) [150]. However, it is possible that simultaneous gene transfer to the brain is required to address all clinical manifestations of Pompe diseases, since there is evidence for accumulation of glycogen in the central nervous system (CNS), and the brain blood barrier (BBB) limits the access of GAA from circulation [151].

#### 6.1.2. Gene Supplementation for Neurological Diseases

The attributes of HC-AdVs, namely the stability of transgene expression and the reduced immunogenicity are particularly important when they are applied in the CNS. In addition, high cloning capacity is often required to allocate large transgenes (such as ion channels), combinations of genes (such as biosynthetic pathways for dopamine production in Parkinson’s disease) or complex regulatory regions to restrict expression to specific cell populations. Parenchymal administration of HC-AdV in rodents (up to 2 × 10^9^ vp) was well-tolerated and avoided the generation of NAbs [152,153,154,155]. After intrathecal administration in rodents and NHP, none or negligible local immune reaction or systemic toxicity have been observed [101,152,156]. Several studies have carried out a complete neuropathological and cell immune infiltration analysis after vector intracranial administration, showing no long-term major changes apart from those associated to the needle tract [153,155]. No signs of inflammation or toxicity were found in peripheral organs such us liver or kidneys (evaluating both histology and enzymatic activity). Haematological and serum biochemical analysis were normal, in contrast with the systemic administration of vectors. No abnormalities were observed in behavioral testing, and animal growth curves were not affected after treatment [153,154]. However, changes in the transcriptome of human midbrain neuroprogenitor cells (hmNPCs) have been observed after infection with HC-AdVs [157]. In general, mild enrichment score was obtained for four main functional gene categories: (1) Cell cycle or DNA damage response, many of them implied in anti-apoptotic functions; (2) trafficking and neuronal remodelling; (3) immune-response; and (4) biochemistry and metabolism. A stronger enrichment was observed in genes implicated in nervous system development 2 h after infection, and in cellular assembly and organization 5 days post-infection. At the same time, a widespread downregulation of genes involved in neuronal development and cell assembly was observed after HC-AdV infection in concordance with previous reports [158]. It is interesting to note that HC-AdV induced slightly minor transcriptome alterations than CAV-2 or lentiviral vectors (495 transcripts modulated *vs* 592 and 728, respectively) and a weaker immune response, although it could be due to a lower transduction rate [157]. It is worthy to mention that the safety of HC-AdVs can be improved by implementing drug-inducible methods to control expression of transgenes, such as the Tet-on transactivation system. Importantly, the FDA has already approved an administration regimen of doxycycline for this purpose [154]. Controlling the amount and duration of transgene expression can be especially important in the brain, where chronic alteration of proteostasis may lead to neurodegeneration. Gene transfer into the CNS can offer advantages too, since intracerebral administration of HC-AdVs confers partial protection from pre-existing systemic anti-Ad immunity [62,64,159,160,161,162,163,164], reducing one of the major drawbacks of AdV vectors in humans. The route of administration plays a crucial role in the safety and efficacy of these vectors in the brain. While a careful intraparenchymal administration elicits negligible systemic cellular or humoral responses, other routes such as intraventricular, meningeal or choroid plexus administration can elicit systemic immune responses [165,166,167,168,169].

As previously mentioned, vectors derived from CAV-2 are especially suited for neuronal transduction and axonal transport, owing to their peculiar docking and intracellular trafficking system [114,170,171,172]. Since a recent review on this subject is available, we will not expand it here [116]. A CAV-2-derived HC-AdV vector encoding the lysosomal enzyme β-glucuronidase was able to improve the neurological status in a mouse model of mucopolysaccharidosis type VII (MPSVII) [115], in which the systemically administered enzyme cannot cross the BBB. Although some discrepancies exist about the percentage of cell populations infected by human HC-AdVs, it is known that they can infect not only astrocytes but also neurons, microglia, oligodendrocytes, and ependymal cells [64,155]. CAR expression is thought to be mainly restricted to neurons in adult mice, *Microcebus murinus* brains and humans [171,173,174,175], although it has been also detected in astrocytes and astrocytic precursors, microglia, choroid plexus, retinal cells [176,177,178,179], and in germinal zones of rodent brains [172,174,178,180,181]. In neurons, CAR can be detected in axons, dendrites, and somas, and in the presynaptic fraction of synaptosome-enriched extracts obtained from adult mouse, *Micronebus murinus*, and human brains [174]. However, it should be taken into account that some studies report little correlation between AdV receptor expression (CAR, integrins or MHCI) and the HC-AdV transduction efficiency or their biological function [182]. Apart from the natural tropism of vectors, re-targeting approaches have led to an increment in transduction of specific cell populations such as the sensory neurons [101,183]. These capsid-modified HC-AdVs showed specificity and efficacy in a moue model of neuropathic pain. Extrapolation of findings obtained in cell culture and animal models to the clinical reality should be done with extreme caution, especially in structures with such complexity and evolutionary divergence as those forming the CNS. So far, HC-AdVs have not been administered to human patients in the brain, but clinical trials using OAVs predict good tolerance [184].

Apart from therapeutic applications, HC-AdVs are an excellent tool for neurosciences and disease modelling in the CNS. They provide an alternative to traditional ablation/injury methods or chemical labelling of neuronal populations. One example is the TRIO technique that combines CAV-2, AAV, and rabies virus vectors to map input–output connections allowing to delineate the brain information trafficking [185]. A moderate retrograde axonal transport of HAdV5 vectors has been documented, opening the possibility of being used in this type of techniques [113,155,186]. Even if recently implanted, opto- and chemogenetic techniques are considered already essential since they elucidate the function of neurons and the circuits in which they are involved. Both technologies often require the use of Cre knock-in mice [187], but the combination of these techniques with Cre-expressing viral vectors could increase their resolution to the cellular level. HC-AdV vectors have been used for modelling inflammation related to neurodegenerative diseases, by overexpression of a mutated form of leucine-rich repeat kinase 2 (LRRK2) in mice [188] and NHPs [175]; and for developing a chronic in vitro model of Huntington´s disease in primary neuronal cultures [189].

Finally, we should be aware that astrocytes could also be targets for gene therapy in neurological diseases, as they interact with other cell types including neurons, microglia, brain microvascular endothelial cells, and ependymal cells throughout the brain, contributing to the disease and recovery processes [190].

#### 6.1.3. Muscle-Directed Gene Supplementation

Although adenoviral transduction in adult skeletal muscle is not as efficient as in liver, HC-AdVs offer the opportunity to deliver the full cDNAs of genes involved in the most frequent muscular dystrophies, which are often too large to be packaged in other viral vectors. This is the case of *dystrophin* (14 Kb), the gene responsible for the devastating Duchenne Muscular Dystrophy (DMD) [191]. A HC-AdV encoding dystrophin under the control of a strong ubiquitous promoter (CAG) achieved restoration of dystrophin expression [37]. Improvement of motor performance and survival have been observed in mouse models of the disease when the vector is injected in different muscle groups [192,193]. Interestingly, efficient transduction in the diaphragm and subsequent amelioration of respiratory function could be achieved by intraperitoneal injection of the vector [194]. Transduction of muscle-related cells, including myoblasts, can be increased by chimeric fibers harboring the HAdV50 shaft and knob [195]. Fiber knob from HAdV3 (5/3 chimera) also increases infection of muscles and allows sustained expression of dystrophin after local administration in mice [196].

#### 6.1.4. Ex-Vivo Gene Supplementation

For diseases in which therapeutic effects can be obtained by secretion of a therapeutic gene into circulation at moderate levels, the TARGT approach (transduced autologous restorative gene therapy) has been proposed. Patient’s dermal fibroblasts are isolated, transduced in vitro with HC-AdVs and implanted subcutaneously. This strategy has been applied for the expression of erythropoietin (Epo) in end-stage renal failure patients. A phase I-II clinical trial demonstrated the feasibility and safety of this treatment. Epo was detected in circulation, and hemoglobin levels were stabilized for at least 5 months after a single cell implantation [197]. In addition, TARGT has been described at the preclinical level for expression of IFN-α [198] and the anti-Her2 monoclonal antibody trastuzumab [199].

### 6.2. Genome Integration and Gene Editing

Episomal maintenance of vector genomes avoids the risk of insertional mutagenesis, but at the same time it precludes the use of standard HC-AdVs when the target cell has an active turnover. Nevertheless, far from being outside the scope of these vectors, HC-AdVs are suitable platforms for stable modification of this type of cells. One possibility to maintain transgene expression is to synchronize vector and host genome replication, without the need for integration. As a proof of concept, the Epstein-Barr virus (EBV) nuclear antigen 1 (EBNA-1) was expressed from a HC-AdV, and sequences acting as origins of replication were incorporated (family of repeats from EBV and a 19 Kb fragment from human chromosome 10). The Cre-loxP system was used to circularize the vector genome in transduced cells. The resulting circular double-stranded DNA molecule was replicated in the S phase of the cell cycle and segregated in daughter cells, maintaining transgene expression for several passages [200].

However, most efforts are recently aimed at gene editing or controlled chromosomal integration. HC-AdVs offer safe and efficient vector/host genome interactions owing to the efficacy of gene delivery into the nucleus, the extended cloning capacity, the genome stability, and the blunted cellular immune responses against transduced cells. Random integration can be obtained using *piggyBack* or *Sleeping beauty* (SB) transposon systems [117]. To this end, the expression cassette flanked by *cis*-acting sequences and the transposase are usually contained in separate vectors. In the case of SB, efficient integration requires circularization of DNA. Therefore, a site-specific recombinase is usually expressed together with the transposase. Random integration and the need of co-delivery are compatible with ex vivo approaches, in which high rate of co-infection and further characterization/selection of cells are feasible [201]. For in vivo approaches, integration into defined genome sequences is preferred. To this end, HC-AdV can accommodate site-specific nucleases such as zinc finger nucleases (ZFN), transcription activator-like effector nucleases (TALEN), and clustered regularly interspaced short palindromic repeats-associated protein 9 (CRISPR/Cas9) [202,203]. Generation of double strand breaks in the host genome favors the integration of the therapeutic DNA sequences following the homologous repair (HR) pathways, especially when the donor DNA is present in the same vector [203]. The deleterious effects of high nuclease expression in packaging cells can be mitigated by the incorporation of miRNA target sites into their 3’UTRs [202]. Importantly, DNA templates with large homology arms can be incorporated in HC-AdV, which increases the efficacy and specificity of integration. However, the non-homologous end joining DNA repair process is usually more efficient than the HR, and this can lead to unwanted insertion and deletions, either in target or off-target regions [203]. Therefore, alternative versions of CRISPR/Cas9 with nickase instead of nuclease activity are being developed [204]. Other approaches use the DNA binding domains of ZFN, TALEN, and CRISPR/Cas9 systems fused with other polypeptides to obtain site-specific transcriptional activators or repressors [205,206]. However, they often require persistent expression of the transcriptional modifier to maintain the effect. In contrast, fusion with base editors can obtain permanent correction of pathogenic mutations [207]. So far, in vivo application has relied on pairs of AAVs or non-viral vectors to deliver the 5.2 Kb-long base editor cDNA [207,208], but HC-AdVs are probably on the way.

One potential drawback for in vivo application of all these strategies is the immunogenicity of the transgene products, especially those derived from prokaryotes such as TALEN and CRISPR/Cas9 [209]. Hopefully, when HC-AdVs are used to deliver these genes into rapidly dividing cells, genome modification will occur rapidly and the expression of the foreign protein will be lost before cytotoxic immune responses can eliminate the daughter cells, as recently described for vectors expressing TALENs and CRISPR/Cas9 [210,211]. In any case, avoidance of nucleases and derivatives would eliminate safety concerns regarding genome integrity and immune reactions. Integration of genetic material and substitution of genomic regions containing mutations can be carried out by HR in the absence of nucleases. Since the frequency is low, it requires in vitro enrichment/selection. HC-AdVs can play a major role in these approaches, because the terminal protein (TP) covalently attached to both genome ends reduces the frequency of random integrations [212]. Using a vector carrying fragments of the cystic fibrosis transmembrane conductance (CFTR) locus, infection of 2 × 10^6^ human induced pluripotent stem cells (iPSC) at MOI 350 obtained up to 144 clones harboring integration of the template DNA after positive selection [213]. In 64% of them the integration occurred in the target site, 32% had random integrations, and less than 5% presented aberrant integrations, in which one of the homology arms (interestingly, the right arm) did not recombine with the predicted genomic sequence. This efficacy is compatible with clinical implementation of ex-vivo gene correction, especially if strategies for negative selection of unintended events are implemented. In this thorough study, a direct correlation between the length of the homology arms and the frequency of site-specific integrations has been reported. Starting from the highest frequency when each homology arm was 11.9 Kb in length, they observed a 30% reduction when one of the arms was 9.5 Kb, and a further 50% reduction with a 5.3 Kb-long arm. Nevertheless, selection of corrected clones was feasible even when the length of the smaller homology arm was 1.3 Kb. These requirements could be influenced by the characteristics of the target region and the genetic defects to be corrected. In a previous study performed in mouse embryonic stem cells (ESC), the minimum homology size to obtain meaningful HR frequencies was 6 Kb, probably because the cells harbored a relatively large insertion (1.6 Kb) rather than point mutations [214]. Based on HR, a promising strategy called GeneRide allows the sustained expression of transgenes under the control of strong endogenous promoters such as the albumin promoter. To this end, the template DNA for integration consists of the sequence preceding the stop codon of the endogenous gene, followed by an autoproteolytic 2A sequence and finally the transgene [215]. Proof of concept for the therapeutic potential of GeneRide has been obtained using AAV vectors, but its efficacy could be enhanced using larger homology regions delivered by HC-AdVs.

Examples with particular clinical relevance for gene integration/editing approaches are the respiratory epithelium and the bone marrow (BM). For sustained amelioration of respiratory manifestations in cystic fibrosis (CF), transduction of basal cells with stem cell properties in the airway epithelium is mandatory. The optimization of delivery routes (intratracheal aerosolization under bronchoscopic guidance), coupled with drugs to open intercellular tight junctions (L-α-lysophosphatidylcholine) has improved the access of HC-AdVs to these cells [216,217]. Once this important barrier has been partially overcome, efforts are now focused on the maintenance of therapeutic gene expression. Al least in vitro, a HC-AdV carrying a TALEN and the CFTR cDNA with 4 Kb homology arms achieved a 5% integration efficiency in the AAVS1 locus in patient-derived cells without positive selection [210]. It is unclear at this moment if this approach could be therapeutic in vivo, but the aforementioned ex-vivo approaches in iPSCs look very promising [213]. Biallelic correction was demonstrated using improved transgenes for selection, but it required sequential infection with different HC-AdV targeting each CFTR allele, and a third step for SB-mediated elimination of the exogenous genes [218].

In the case of BM, the development of HC-AdVs based on HAdV35 has increased the transduction efficacy in HSCs and the feasibility of gene editing approaches for a wide variety of diseases, as described in detail in a recent review [219]. Of note, expression of nucleases in these cells could reduce their repopulation potential. This could be avoided by expressing inhibitory peptides, although it requires an additional transduction step [220]. For the treatment of β-globinopathies such as β-thalasemia and sickle cell anemia, reactivation of the fetal γ-globin gene expression can be therapeutic. To this end, disruption of the recognition sequence for a repressor in the γ-globin gene can be obtained using a chimeric 5/35++ HC-AdV encoding a CRISPR/Cas9, without the need of HR. An increase in γ-globin expression was observed using a standard ex-vivo approach [221]. More importantly, similar results were obtained in this study when a modified vector was administrated intravenously, providing the proof of concept that gene editing is feasible in vivo. Instead of pre-conditioning of the host, HSCs were mobilized by G-CSF/AMD3100 treatment. Selective advantage of gene-modified cells was obtained by incorporating the mgmt^P140K^ transgene, which confers resistance and proliferation stimulus in response to O^6^-BG/bis-cloroethyl-nitrosourea (BCNU) treatment. Alternatively, the γ-globin gene has been integrated into the AAVS1 locus by co-administration of a pair of HC-AdVs encoding a CRISPR/Cas9 and the DNA template [222].

The BM can be used as a secretory organ for therapeutic proteins such as coagulation FVIII. In this case, one vector carried the *F8* cDNA together with the mgmt^P140K^ selection gene, and another one expressed the SBx100 transposase to promote integration into the host genome. When these vectors were administered in vivo, the levels of FVIII in circulation reached 5% normal values in a mouse model [223]. Although this level is still lower than that obtained after liver-directed gene therapy [119], permanent transduction of HSCs opens the possibility of local production of therapeutic proteins in the CNS through migration of glial precursors. This would avoid the need to cross the BBB in LSD and other diseases [224].

In some genetic muscular dystrophies such as DMD, early intervention and stable expression of therapeutic genes in myoblasts is important. Some strategies aimed at dystrophin cDNA integration consist of hybrid AdV-AAV vectors, in which integration occurs at the AAVS1 locus in the presence of the AAV Rep protein, as demonstrated in vitro [225]. An alternative for in vivo gene editing in DMD would be the re-setting of reading frames, the alteration of splicing acceptors or the removal of mutated exons by expression of site-specific nucleases. Proof of concept about the restoration of dystrophin expression has been generated in muscle cell populations in culture using E1-E2-deleted AdVs encoding a combination of CRISPR/Cas9 and TALENs [226]. The use of HC-AdV could improve the in vivo performance of this strategy.

### 6.3. Cancer

After many disappointing trials, it is clear that current gene therapy technology does not allow transduction of all the cells in a tumor. However, many immunotherapy approaches can benefit from the properties of HC-AdVs, especially when the expression of transgenes should be placed under the control of drug-inducible systems to avoid toxicity, and when a combination of transgenes is needed. The HC-Ad-TK/TetOn-Flt3L vector encodes the suicide gene thymidine kinase (TK) and the dendritic cell chemoattractant FMS-like tyrosine kinase 3 ligand (Flt3L) under the control of a tet-on inducible system [154,164,227]. This vector has demonstrated promising results in rodent models of glioblastoma. A liver-specific, mifepristone-induced expression system [228] has been used to control expression of interleukin-12 for the treatment of colorectal cancer hepatic metastases, showing tumor eradication in synergy with chemotherapy [229]. This vector was safer than an OAV expressing the same transgene [230]. To further reduce systemic exposure to this toxic cytokine, a fully humanized mifepristone-inducible system controlled by the ubiquitous elongation factor 1α (*EF1α*) promoter was developed [231]. The vector carrying this expression cassette can be administered intratumorally, maintaining its antitumor effect in pancreatic cancer models. HC-AdVs can also be employed to express tumor antigens in dendritic cells (DC). The absence of viral genes in the vector preserves the T-cell stimulating capacity of transduced DCs, obtaining better antitumor effects than FGAdVs encoding the same antigen [103].

### 6.4. Vaccination and Therapy against Infectious Diseases

The strong innate immune responses against AdV particles can be exploited for the design of genetic vaccines. As introduced in the previous sections, some evidences indicate that HC-AdVs outperform FGAdVs when they express the same antigen. This is in part due to the cell cycle arrest caused by FGAdVs in DCs [103,232]. In addition, de novo expression of adenoviral genes, which are usually immunodominant, is detrimental for the generation of multi-specific T-cell responses against the target antigen. This concept was demonstrated when both types of vectors were used for the expression of hepatitis B virus small surface antigen (HBsAg) [233], or the merozoite surface protein 1 (MSP-1) from *Plasmodium falciparum* for vaccination against malaria [234]. Similar results were observed when vectors encoded a model antigen (β-galactosidase) [235]. In this case, the stimulation of both humoral and cellular immune responses was more efficient with the HC-AdV. Interestingly, ex vivo transduction of DCs instead of intraperitoneal administration achieved similar results, while anti-adenoviral responses were reduced. For the clinical translation of these approaches, it should be taken into account the high prevalence of immunity against certain HAdV types in the adult population, including the prototypic HAdV5. This not only compromises the efficacy of transgenic antigen expression, but also boosts immune responses against the adenoviral capsid proteins. In a clinical trial of a HAdV5-based vaccine against HIV, higher risk of infection was observed in a subgroup of vaccinated patients pre-exposed to HAdV5 [236]. Although the mechanism is still controversial [237], one hypothesis is that mucosal homing of anti-HAdV5 immune cells made subjects more susceptible to HIV infection. To overcome these problems, HVs derived from other human and animal AdVs can be employed to produce the HC-AdVs [103]. The experience gained with FGAdVs and replication-competent AdVs, including clinical trials, indicate that HAdV4, HAdV35, HAdV26, and chimpanzee AdV3, Ad7, AdV63, AdV68, among others, are promising candidates as vector platforms [238,239,240,241,242,243]. The current Covid-19 pandemic has boosted the interest on genetic vaccines, and some human and simian AdV vectors encoding the SARS-Cov-2 spike protein are advanced candidates (https://cen.acs.org/pharmaceuticals/vaccines/Adenoviral-vectors-new-COVID-19/98/i19). These approaches are based on previous experimental vaccines against MERS-CoV [244,245]. Other anti-viral strategies using HC-AdVs include the controlled expression of cytokines such as IFNα [246] and IL-12 [247] against HCV and HBV, respectively. However, evaluation in stringent animal models for both diseases revealed limited therapeutic effect. Finally, a HC-AdV encoding a CRISPR/Cas9 system with multiple guides against the hepatitis B genome achieved reduction of HBsAg expression and viral copies in liver-derived cell lines [248]. Further work should determine the efficacy of this approach in animal models of HBV infection.

## 7. Conclusions

During the past two decades, a vast number of preclinical studies have demonstrated the ability of HC-AdVs to transduce relevant target cells in vivo, especially hepatocytes, neurons and endothelial cells. Stable episomal maintenance of genomes in these cells, resulting in sustained expression of transgenes, has been demonstrated not only in rodents but also in larger animal models, including NHPs. Despite clear evidence of therapeutic effect in a large number of disease models, clinical experience with these vectors is virtually absent. This is in part because translational efforts have been focused in other vector platforms, such as AAV. When therapeutic cassettes can fit into the 4.5 Kb size constraint of AAVs, these vectors have shown similar pre-clinical results than HC-AdVs, but they elicit less inflammatory responses and are easier to produce under GMP conditions. However, the field of HC-AdVs is making progress in delivery routes, methods to prevent and manage side effects, and protocols for large-scale production. We believe these vectors will play an important role in the gene therapy arsenal in the near future. Not only as an alternative to AAVs, but more importantly, covering unmet needs in the fields of gene editing and the transfer of large sequences.

## Figures and Tables

**Table 1:** Characteristics of all human adenovirus (HAdV) types, and selected examples of animal AdVs adapted as gene therapy vectors.

Genus	Natural Host	AdV Species (Types)	Receptors	Tropism	Genome Size (Kb)
**Mastadenovirus**	Human (HAdV)	A (12, 18, 31,61)	CAR, INT	Epithelium (respiratory, intestinal)	34–36
		B (**3**, **7**, **11**, 14, 16, 21, 34, 35*, **50**, 55)	CD46, DSG2, CD80, CD86, LPR, INT	Epithelium (respiratory, ocular, urinary); lymphoid, HSC	
		C (1*,2*,5*,6*, 57)	CAR, HSPG, LPR, MHC-I, SR, VCAM-1, INT	Epithelium (respiratory, ocular, intestinal); liver	
		D (8–10, 13, 15, 17, **19**, 20, 22–25, **26**, 27, **28**, 29, 30, 32, 33, 36–39, 42, **43**, 44–47, **48**, **49**, 51, 53, 54, 60a, 62–65, 67, 69, 71, 81)	SA, CD46, CAR, INT	Epithelium (respiratory, ocular, intestinal)	
		E (**4**)	CAR, INT	Epithelium (respiratory, ocular)	
		F (40, **41**)	CAR	Epithelium (intestinal)	
		G (52)	SA, CAR	Epithelium (intestinal)	
	Canine (CAdV)	A (2*)	CAR	Epithelium (respiratory); neurons	31
	Simian (SAdV) Chimp.	C (**3**, **Pan3**)	CAR	Epithelium (respiratory, ocular, intestinal); liver	36
		E (**7**, **63**, **68**)	CAR	Epithelium (respiratory, ocular, intestinal); liver	36.5
	Porcine (PAdV)	A (3*)		Epithelium (respiratory, intestinal)	34
	Murine (MAdV)	A (**1**)	INT, HSPG	Epithelium (respiratory, ocular); brain, spinal cord, spleen	31
	Bovine (BAdV)	A (**1**)		Epithelium (respiratory)	35
		B (**3**)	SA	Epithelium (respiratory, intestinal); liver, kidney, heart	34.4
**Aviadenovirus**	Fowl (FAdV)	A (CELO) (**1**)	CAR	Epithelium (respiratory); liver	43.8
		C (**4**, **10**)	CAR	Epithelium (respiratory); liver	45.6
		D (**9**)		nd	45
		E (**8**)		Epithelium (respiratory); liver	45
**Atade-novirus**	Ovine (OAdV)	D (**7**)	INT	Epithelium (respiratory, intestinal)	29.6

Viruses adapted as gene therapy vectors are marked in bold, and those with HC-AdV versions are distinguished by an asterisk. The best characterized receptors are described (with primary receptor underlined), but they are not exclusive. Note: for chimpanzee AdV we have used the classification described in [16], but some of them can be included in human *Mastadenovirus* species. CAR, Coxackie and Adenovirus receptor; DSG2, desmoglein 2; HSPG, heparan sulphate proteoglycans; INT, integrins; LPR, low-density lipoprotein receptor related protein; MHC-I, major histocompatibility complex-I; SA, syalic acid; SR, scavenger receptor; VCAM-1, vascular cell adhesion molecule-1.

## Abbreviations

A1AT	Alpha 1 anti-trypsin
A1ATD	Alpha 1 anti-trypsin deficiency
AAV	Adeno-associated virus
AAVS1	Adeno-associated virus integration site 1
ABCA1	ATP-binding cassette subfamily A member 1
AdV	Adenovirus
AGT	Alanine:glyoxylate aminotransferase
AIP	Acute intermittent porphyria
ALT	Alanina aminotrasferasa
BBB	Blood brain barrier
BCNU	Bis-cloroethyl-nitrosourea
BM	Bone marrow
CAR	Coxsackie and adenovirus receptor
CAV-2	Canine adenoviral vector type 2
CF	Cystic fibrosis
CFTR	Cystic fibrosis transmembrane conductance regulator
ChAdV	Chimpanzee adenovirus
CNS	Central nervous system
CPS1D	Carbamoyl phosphate synthetase 1 deficiency
CR1	complement receptor 1
CRISPR/Cas	Clustered regularly interspaced short palindromic repeats-associated protein 9
DC	Dendritic cells
DMD	Duchenne muscular dystrophy
EBNA-1	Epstein-Barr nuclear antigen 1
EBV	Epstein-Barr virus
EF1α	Elongation factor 1 alpha
Epo	Erythropoietin
ESC	Embryonic stem cells
FGAdV	First-generation adenoviral vector
FLP	Flippase
Flt3L	FMS-like tyrosine kinase 2 ligand
FRT	Flippase recognition target
GAA	Acid alpha-glucosidase
GMP	Good manufacturing practices
HAdV	Human adenovirus
HBsAg HBV	Hepatitis B virus small surface antigen Hepatitis B virus
HC-AdV	High-Capacity adenoviral vector
HCV	Hepatitis C virus
HD-AdV	Helper-Dependent adenoviral vector
HDL	High-density lipoprotein
hmNPC	Human midbrain neuroprogenitor cells
HR	Homologous recombination
HSC	Hematopoietic stem cells
HSPG	Heparan sulphate proteoglycans
HSV	Herpes simplex virus
HV	Helper virus
HVR5	Hypervariable region V
IFN	Interferon
IgM	Immunoglobulin M
IL-6	Interleukin 6
iPSC	Induced pluripotent stem cells
ITR	Inverted terminal repeats
iu	Infection units
LDL	Low density lipoprotein
LDLR	Low density lipoprotein receptor
LRRK2	Leucine-rich repeat kinase 2
LSD MERS-CoV	Lysosomal storage disorders Middle East respiratory syndrome coronavirus
MHCI	Major histocompatibility complex class I
MOI	Multiplicity of infeciton
MPSVII	Mucopolysaccharidosis type VII
MSP-1	Merozoite surface protein 1
NAb	Neutralizing antibody
NHP	Non-human primates
OAV	Oncolytic adenovirus
PBGD	Porphobilinogen deaminase
PEI	Polyethylene glycol
PEPCK	Phosphoenolpyruvate carboxykinase
pfu	Plaque forming units
PH1	Primary hyperoxaluria type 1
PRR	Pattern recognition receptor
pTP	Pre-terminal protein
SAdV SARS-CoV-2	Simian adenovirus Severe acute respiratory syndrome coronavirus 2
SB	Sleeping beauty
TALEN	Transcription activator-like effector nucleases
TARGT	Transduced autologous restorative gene therapy
TLR	Toll-like receptor
TNFα	Tumor necrosis factor alpha
TP	Terminal protein
TRIO	Tracing the relationship of inputs and outputs
UGT1A1	Uridine diphospho-glucuronyl transferase 1A1
vg	viral genomes
VLDL	Very low density lipoprotein
vp	viral particles
ZFN	Zinc Finger Nucleases
